# Microstructural Stability and Densification Behavior of Cantor-Type High-Entropy Alloy Processed by Spark Plasma Sintering

**DOI:** 10.3390/ma18194625

**Published:** 2025-10-07

**Authors:** Marcin Madej, Beata Leszczyńska-Madej, Anna Kopeć-Surzyn, Paweł Nieroda, Stanislav Rusz

**Affiliations:** 1 Faculty of Metals Engineering and Industrial Computer Science, AGH University of Krakow, 30 Mickiewicza Ave, 30-059 Krakow, Poland; kopecsurzyn@agh.edu.pl; 2 Faculty of Non-Ferrous Metals, AGH University of Krakow, 30 Mickiewicza Ave, 30-059 Krakow, Poland; bleszcz@agh.edu.pl; 3 Faculty of Materials Science and Ceramics, AGH University of Krakow, al. Mickiewicza 30, 30-059 Krakow, Poland; pnieroda@agh.edu.pl; 4 Department of Manufacturing Technology, Faculty of Mechanical Engineering, VSB—Czech Technical University in Prague, Technická 4, 166 07 Praha 6, Czech Republic; stanislav.rusz@fs.cvut.cz

**Keywords:** high entropy alloys, Cantor alloy (CoCrFeMnNi), spark plasma sintering (SPS), densification behavior, microstructural evolution, powders

## Abstract

High-entropy alloys (HEAs) of the Cantor type (CoCrFeMnNi) are widely recognized as model systems for studying the relationships between composition, microstructure, and functional performance. In this study, atomized Cantor alloy powders were consolidated using spark plasma sintering (SPS) under systematically varied process parameters (temperature and dwell time). The densification behavior, microstructural evolution, and mechanical response were investigated using Archimedes’ density measurements, Vickers hardness testing, compression tests, scanning electron microscopy, and EDS mapping. The results reveal a non-linear relationship between sintering temperature and densification, with maximum relative densities obtained at 1050 °C and 1100 °C for short dwell times. Despite the ultrafast nature of SPS, grain growth was observed, particularly at elevated temperatures and extended dwell times, challenging the assumption that SPS inherently limits grain coarsening. All sintered samples retained a single-phase FCC structure with homogeneous elemental distribution, and no phase segregation or secondary precipitates were detected. Compression testing showed that samples sintered at 1050 °C and 1070 °C exhibited the highest strength, demonstrating the strong interplay between sintering kinetics and grain cohesion.

## 1. Introduction

High-entropy alloys (HEAs) constitute one of the most innovative classes of metallic materials, attracting significant attention from both scientific and industrial communities in recent years [1,2,3]. Their uniqueness lies in the design of chemical composition, where several principal elements are present in nearly equimolar proportions, leading to the formation of high-entropy phases with simplified crystalline structures [4,5]. These materials exhibit exceptional mechanical, thermal, and corrosion resistance properties, often surpassing those of traditional engineering alloys [6,7]. HEAs, particularly the Cantor alloy (CoCrFeMnNi), remain among the most recognized and extensively studied representatives within this group [8]. Since its discovery, the Cantor alloy has attracted significant scientific attention due to its exceptional mechanical properties, distinguishing it from both conventional and other HEAs [9,10]. It exhibits a single-phase FCC structure with excellent ductility and resistance to brittle fracture, even under cryogenic conditions [9,10,11]. A notable characteristic of the Cantor alloy is its strain hardening capability, which imparts high strength while retaining excellent plasticity [12,13]. The alloy achieves elongations to failure in the range of 60–70% and tensile strengths exceeding 1 GPa, with fracture toughness surpassing 200 $MPa\sqrt{m}$ at low temperatures [11,14]. Moreover, its high corrosion resistance and microstructural stability have established it as a reference material in HEA research and a foundation for designing new alloys with balanced properties [15,16]. Despite the rapid development of novel HEA compositions, the Cantor alloy remains scientifically and practically valuable, serving as a model for the optimal compromise between strength, ductility, and resistance to extreme conditions [8,9,17].

Among the manufacturing methods for HEAs, powder metallurgy coupled with spark plasma sintering (SPS) plays an increasingly important role [1,4,18,19]. SPS is a technique enabling rapid and efficient densification of metallic powders by applying simultaneous pulsed electric current and mechanical pressure [1,4]. This method allows for the production of dense materials with fine microstructures and controlled mechanical properties, while significantly reducing sintering time and grain growth [2,3]. Current research on SPS sintering of HEAs focuses on optimizing process parameters such as temperature, pressure, sintering time, and heating rate [5,20,21]. Precise control of these parameters facilitates achieving optimal density, uniform microstructure, and desired mechanical characteristics such as hardness, tensile strength, and creep resistance [3,21]. Another key trend is the control of microstructure and phases formed during the SPS process [4,6,18]. Rapid heating and short sintering times allow for the development of nanocrystalline or ultrafine-grained structures, which greatly influence the functional properties of the materials [5,7]. Researchers emphasize that controlling grain size and distribution of secondary phases is critical for achieving high strength and wear resistance [3,6]. Increasingly, the literature reports the use of powder composites and reinforced powders in SPS processing [5,22]. Adding ceramic particles such as carbides or nitrides enables the production of HEA composites with enhanced wear, corrosion, and oxidation resistance [6]. Such materials are promising for applications in tooling, energy, and aerospace industries, where materials must withstand severe operating conditions [4,7].

Particular attention has been paid to HEAs containing refractory elements such as Cr, Mo, Nb, W, and V [18,20]. These alloys exhibit high thermal stability and oxidation resistance, making them suitable candidates for aerospace and energy applications, where materials are exposed to high temperatures and aggressive environments [6,19]. For example, CrMoNbWV-type alloys combine strength and oxidation resistance favorably [20].

The advancement of SPS technology aligns with current environmental and sustainable development trends [1,4,7]. Through reduced sintering time and lower temperatures, SPS is considered an environmentally friendlier technology compared to traditional sintering methods [2,4,21]. Additionally, precise control over microstructure and properties enables the design of alloys with increased lifespan and resistance to wear, translating into reduced material and energy consumption throughout the product lifecycle [1,5,6].

Despite significant progress, challenges persist in fully controlling microstructure and phase homogeneity in large components, as well as modeling diffusion processes and sintering kinetics [5,18,20]. Extensive research continues on understanding the effects of alloying additions and process parameters on final material properties [3,5,22]. The literature stresses that further development in this field requires multidisciplinary collaboration incorporating metallurgy, materials engineering, and physicochemical process modeling [6,7].

In summary, current trends in SPS sintering of HEAs involve process parameter optimization, microstructure and phase control, composite development, and industrial application implementation [4,5,19]. SPS remains one of the most promising fabrication technologies for HEAs, enabling the production of materials with unique properties and broad engineering applications [3,6,20].

The novelty of this work lies in the systematic investigation of densification behavior, microstructural evolution, and mechanical properties of atomized Cantor alloy powders consolidated by spark plasma sintering under varied temperature and dwell time conditions. This study reveals a non-linear relationship between sintering temperature and relative density, and notably documents grain growth during the ultrafast SPS process, challenging the common assumption that SPS inherently suppresses grain coarsening. Additionally, the retention of a homogeneous single-phase FCC structure without phase segregation, paired with enhanced mechanical strength, emphasizes the strong interplay between sintering kinetics and microstructural stability, which has significant implications for the processing of high-entropy alloys.

## 2. Materials and Methods

The CoCrFeMnNi prealloyed powder used in this study was supplied by Luoyang Tongrun Info Technology Co., Ltd. (Luoyang, China). Its chemical composition and properties are presented in Table 1 and Table 2.

**Figure 1:**
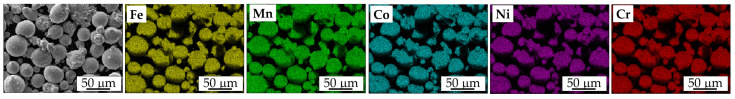
Morphology of the HEA powder particles and elemental distribution maps of Fe, Mn, Co, Ni, and Cr acquired by EDS analysis, SEM.

The morphology of the powder used for the research are shown in Figure 1.

The CoCrFeMnNi alloy powder used in this study has an approximately spherical shape (Figure 1). Deviations from this shape result from the adhesion of ultrafine particles to larger droplets that are still solidifying in the atomization chamber. As seen in Figure 1, particles with attached finer grains constitute the majority. Elemental distribution maps presented in Figure 1 confirm a homogeneous distribution of the constituent elements within the powder particles. Although this particle morphology appears suitable for SPS sintering. HEA Cantor’s alloy powders were directly loaded into graphite POCO EDM-1 dies without prior compaction and sintered using the SPS technique (RACS25 hybrid device from Materials Design Systems and Devices LLC, Kraków, Poland). Cylindrical samples with dimensions of Ø10 × 5 mm were produced. Sintering was performed under variable process conditions, which were set close to the previously determined optimal sintering temperature of 1050 °C, for 7.5 min, under a uniaxial pressure of 50 MPa [23].

The schematic of the sinter manufacturing process is presented below in Table 3:

Interestingly, the compacts sintered at 1090 °C exhibited flashing, which led to their exclusion from further studies; shortening the sintering time to 3 min did not prevent this. Increasing the temperature to 1100 °C did not cause such negative effects, so the sinters obtained at this temperature were used for further investigations.

The density of the sintered samples was determined using the Archimedes method by weighing them in water and air, without the need for saturation. Microhardness and hardness measurements were performed using a Vickers hardness tester (INNOVATEST, Maastricht, The Netherlands) according to the ISO 6507-1:2023 standard [24]. Microstructural examinations were carried out by scanning electron microscopy (SEM) employing a Schottky-type electron gun (SU-70, Hitachi Ltd., Tokyo, Japan), equipped with a NORAN System 7 (Waltham, MA, USA) X-ray microanalysis system (EDS). The average grain diameter was determined using ImageJ V.1.54k software [25], with approximately 50 grains measured per sample to ensure representativity.

Subsequently, static uniaxial compression tests were performed at room temperature using a Zwick Roell Z020 testing machine. For this purpose, sets of four cylindrical samples, each with a height-to-diameter ratio of 1.5 (D = 3 mm, h = 4.5 mm), were prepared for each variant according to ASTM E-9 [26]. The compression test was carried out at a strain rate of 8 × 10^−3^ s^−1^ and was stopped when a permanent strain of 50% was reached. Three samples from each material were tested in the static compression regime.

## 3. Results

**Figure 2:**
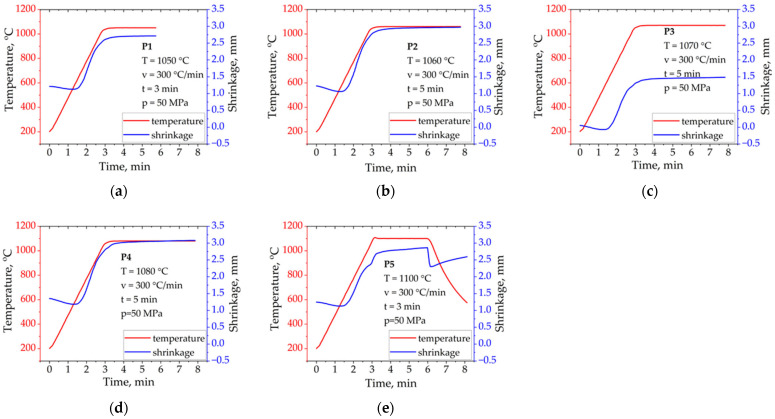
Shrinkage curves of the Cantor high-entropy alloy: (**a**) T = 1050 °C, t = 3 min; (**b**) T = 1060 °C, t = 5 min; (**c**) T = 1070 °C, t = 5 min; (**d**) T = 1080 °C, t = 5 min; (**e**) T = 1100 °C, t = 3 min.

In the present study, the optimal spark plasma sintering (SPS) parameters for the investigated alloy were established based on systematic experiments, in which material density and microstructural stability were adopted as the main criteria. Sintering tests were performed over a temperature range of 875–1100 °C in 25 °C increments, under a constant punch pressure of 50 MPa. The experimental results indicate that at 50 MPa, the most favorable sintering temperature was 1050 °C, yielding a nearly fully dense material with minimal porosity. These findings further confirm the high efficiency of the SPS technique, allowing for the fabrication of dense solids at relatively low temperatures and significantly reduced processing times. Additionally, the rapid densification and short exposure to elevated temperatures effectively limited grain growth in the resulting material [23]. In Figure 2, the sintering curves obtained for various processes are presented. The experiments were conducted under process parameters that were either optimal or in close proximity to the optimal sintering conditions determined in this study. These curves provide insight into the densification behavior of the material under different SPS parameters.

The shrinkage behavior of the Cantor high-entropy alloy during Spark Plasma Sintering (SPS) exhibits distinct kinetics characteristic of this process, as shown in Figure 2. Curve (a) corresponding to 1050 °C with a 3 min dwell time displays a rapid initial shrinkage within approximately the first 1.5 min, indicative of quick neck formation and pore closure. However, the short dwell time likely limits further grain boundary migration—a mechanism that is typically difficult to activate during SPS—resulting in a relatively lower final densification. Curve (b), measured at 1060 °C with a 5 min hold, reveals a more extended densification process featuring an initial rapid shrinkage phase followed by a slower continuation. This reflects enhanced grain boundary mobility and improved final density due to longer thermal exposure, although grain boundary migration during SPS may still be kinetically constrained. At 1070 °C and 5 min (curve c), a similar densification pattern is observed but with a greater total shrinkage, indicating more effective diffusion mechanisms and microstructural development, despite the inherent difficulty of grain boundary migration in SPS processes. Curve (d) recorded at 1080 °C with 5 min exhibits further improvement in densification and microstructural evolution, as evidenced by a larger shrinkage magnitude compared to lower temperatures. In contrast, curve (e), corresponding to the highest temperature of 1100 °C but with a reduced dwell time of 3 min, shows a sharp initial shrinkage followed by a plateau phase. This behavior suggests that despite increased temperature, the short hold time kinetically restricts further densification by limiting grain boundary migration and grain growth, which are likely already challenging to activate under SPS conditions.

Overall, the comparison indicates that while longer dwell times at moderate to high temperatures promote more continuous and higher shrinkage levels through activation of diffusion-controlled mechanisms, grain boundary migration—a key factor for densification—is likely hindered under SPS, particularly with shorter dwell times, thereby limiting complete densification even at elevated temperatures. Further microstructural investigations are necessary to precisely determine the relationships between sintering parameters and the densification mechanisms.

**Figure 3:**
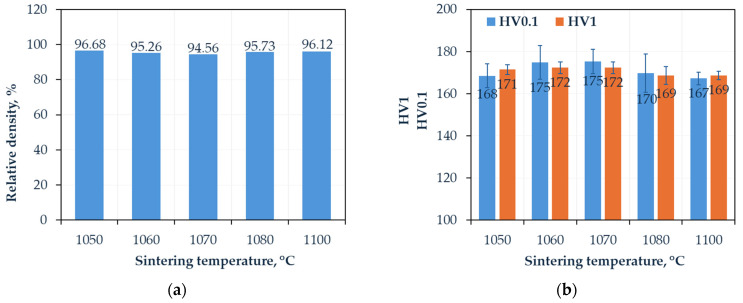
(**a**) Relative density and (**b**) hardness and microhardness of the Cantor high-entropy alloy as a function of FAST/SPS sintering temperature.

The density values obtained by the Archimedes method relative to the theoretical density of the Cantor alloy are presented in Figure 3, together with the hardness and microhardness measurements performed using the Vickers method.

The analysis of the relative density of the Cantor alloy sintered by the FAST/SPS method (Figure 3a) reveals an atypical, non-linear dependence of density on the sintering temperature. The highest density values are observed for samples sintered at 1050 °C and 1100 °C, both subjected to a short dwell time of 3 min, whereas samples sintered at intermediate temperatures (1060 °C to 1080 °C) exhibit a slight decrease in relative density.

This phenomenon can be interpreted in light of the microstructural characteristics of high-entropy alloys, where processes such as grain growth or recrystallization may intensify within this temperature range, inhibiting diffusion mechanisms and thereby hindering densification. Conversely, the lower temperature of 1050 °C, previously determined as optimal in the preliminary study [23], facilitates the greatest densification; however, the short isothermal holding time clearly limited achieving higher density values. Interestingly, the highest final temperature of 1100 °C also promotes improved densification, though the density remains somewhat lower than at the optimal temperature.

Observing the densification curve in Figure 2e, it is evident that following an initial rapid densification phase during the first 2 min of isothermal holding, a significant density drop occurs, followed by a subsequent increase upon cooling. This behavior suggests overheating of grain boundaries, resulting from the nature of electrical pulse interactions at these boundaries. Such effects may cause phase disruption, localized melting, or even vaporization of material—phenomena typical in classical sintering processes [27,28]. However, during SPS sintering, condensation may not occur uniformly on adjacent grains but rather on adjacent surfaces, such as graphite mold walls. Despite these challenges, the applied uniaxial pressure helps achieve relatively high densities.

The hardness and microhardness measurements performed using the Vickers method (Figure 3b) demonstrated that the values are similar regardless of the sintering conditions. Typically, microhardness is slightly higher than hardness, which indicates the effect of porosity influencing the bulk hardness of the material. The range of measured microhardness values (HV0.1) falls between 167 and 175, while for hardness (HV1) the values are between 169 and 172. This shows exceptional consistency of the results, which also typically lie within measurement error margins. Based on this distribution, it can be stated that the electrical phenomena occurring during sintering, when the temperature is changed by 50°, do not induce drastic microstructural changes, such as the decomposition of a single-phase alloy structure into new types of precipitates or the evaporation of individual constituents leading to changes in the alloy’s chemical composition. The sample sintered under optimal conditions exhibits a hardness of 180 HV1, indicating that extending the sintering time to 5 min enables achieving a density above 99%, which further contributes to an increase in hardness.

According to the literature, the Cantor alloy (CoCrFeMnNi) consolidated via Spark Plasma Sintering (SPS) exhibits moderate hardness, which depends on processing parameters and potential surface modifications. Li et al. [29] performed SPS at 950 °C for 5 min, obtaining an initial hardness of 181 HV, with the material reaching a fully dense or near fully dense state. In the study by Liu et al. [30], additional plasma nitriding of SPS-consolidated samples (873 K, 15 h, 600 Pa) increased the surface hardness to 290 HV, representing an enhancement of over 60% compared to the initial value of approximately 180 HV, while preserving the core microstructure. Furthermore, Kumar et al. [31], in a review of SPS processing for high-entropy alloys (HEAs), reported typical hardness ranges for SPS-consolidated Cantor-type materials between 180 and 220 HV, depending on alloy composition and sintering conditions.

An explanation of these phenomena will undoubtedly be provided by the microstructural analysis of the sintered samples obtained under varying conditions. The figure below presents images of the microstructures observed at different magnifications using a scanning electron microscope.

**Figure 4:**
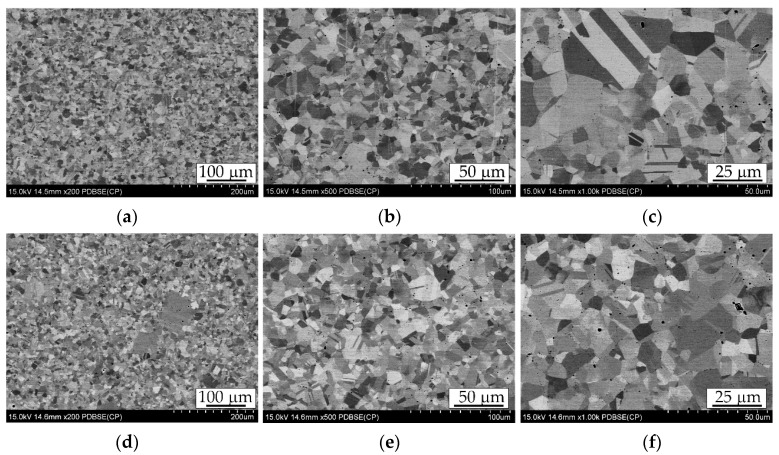

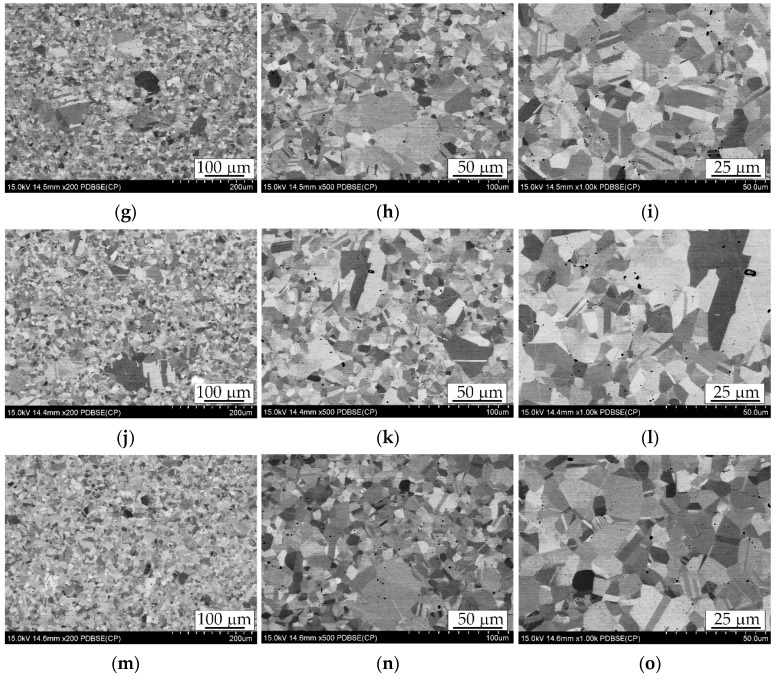
Microstructure of the Cantor high-entropy alloy sintered under different conditions: (**a**–**c**) T = 1050 °C, t = 3 min; (**d**–**f**) T = 1060 °C, t = 5 min; (**g**–**i**) T = 1070 °C, t = 5 min; (**j**–**l**) T = 1080 °C, t = 5 min; (**m**–**o**) T = 1100 °C, t = 3 min. Images taken at different magnifications: (**a**,**d**,**g**,**j**,**m**) 200×; (**b**,**e**,**h**,**k**,**n**) 500×; (**c**,**f**,**i**,**l**,**o**) 1000×. SEM images.

All investigated sinters of the Cantor high-entropy alloy, presented in Figure 4 in order of increasing temperature, are characterized by a single-phase structure, in this case an austenitic (FCC) structure with clearly defined grain boundaries. The interiors of many grains are intersected by annealing twin bands, typical for materials with a face-centered cubic structure [8,32]. The darker, irregularly shaped and unevenly distributed characteristic areas visible in the micrographs are mostly pores; since no secondary phases or impurities were detected in the sintered materials after the sintering process that were present in the initial powder.

The main observed difference is the grain size, which is clearly influenced by the sintering temperature and, to a lesser extent, by the duration of isothermal holding at that temperature. This finding partially contradicts claims often presented in promotional materials regarding the SPS method, which suggest minimal grain growth due to the short exposure at elevated temperatures. Indeed, while the exposure time in SPS is significantly shorter than in traditional sintering—where temperature ramp-up and the sintering process itself usually take much longer—our results show that both temperature and holding time can notably affect grain growth [33,34]. However, the exact temperature at the grain boundaries is not fully known, making it difficult to precisely control the conditions within the grain. This uncertainty leads to an increased grain growth rate, especially in materials where there are no precipitates or foreign phase particles at the grain boundaries to inhibit their growth. A closer examination of the grain size results, summarized in Table 4, reveals the measurements obtained using the ImageJ software.

The average grain size in the investigated sintered samples ranges from 8.74 to 10.59 μm, with the smallest average grain size observed in the sample sintered at 1060 °C for 5 min, and the largest at 1080 °C for 5 min. It was found that both a combination of high temperature with longer sintering time (e.g., 1070 and 1080 °C for 5 min) and elevated temperature with shorter dwell time (1100 °C for 3 min) promote grain growth, adversely affecting the microstructure.

The finest microstructure was obtained either at moderate temperatures with longer sintering times (1070–1080 °C, 5 min) or at the highest temperature with the shortest sintering time (1100 °C, 3 min). Analysis of locally observed large grains revealed the greatest grain growth in the sample sintered at 1080 °C, where large grains reached up to 114.27 μm, coinciding with the highest average grain size. Conversely, the smallest large grains were found in the sample sintered at 1050 °C (51.00 μm), despite this sample showing the second largest average grain size. In the other samples, the size of large grains tended to increase with both sintering temperature and time. Abnormal grain growth during spark plasma sintering results from localized heating, rapid heating rates, and simultaneous application of pressure, which create temperature gradients and stress fields. These conditions promote preferential growth of certain grains, especially when sintering parameters exceed optimal values, leading to non-uniform grain size distribution and larger grains dominating the microstructure.

The presence of these large grains disrupts the uniformity of the microstructure and may contribute to deterioration in the mechanical properties of the material. Therefore, minimizing localized grain growth is critical for achieving a desirable alloy structure.

Observations of twins indicate that their number and development increase with grain size. In samples sintered at lower temperatures (1050, 1060 °C), twins are shorter and less frequent, whereas in samples sintered at higher temperatures and longer times (1070, 1080 °C), they occur abundantly and often form multiple bands. This results from the fact that larger grains in the FCC (RSC) structure exhibit a higher probability of annealing twin formation [35], which confirms the stability of the austenitic phase in the studied alloy.

However, a key feature of functional HEAs is also the uniform distribution of each element (chemical homogeneity) within the microstructure, which has a decisive influence on the mechanical and physical properties of the material, such as corrosion resistance and hardness [36,37].

**Figure 5:**
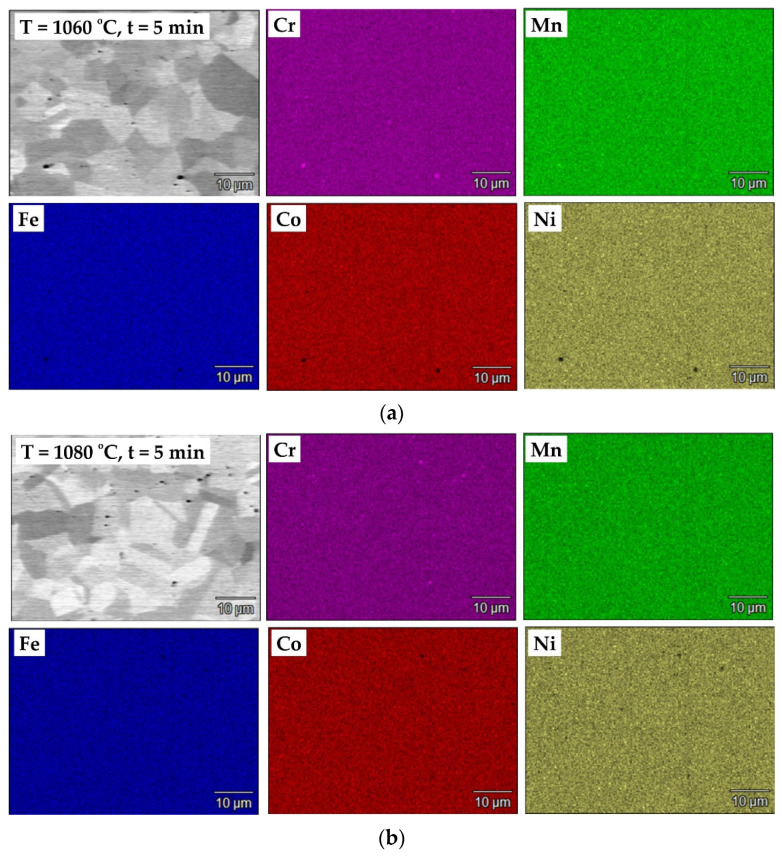

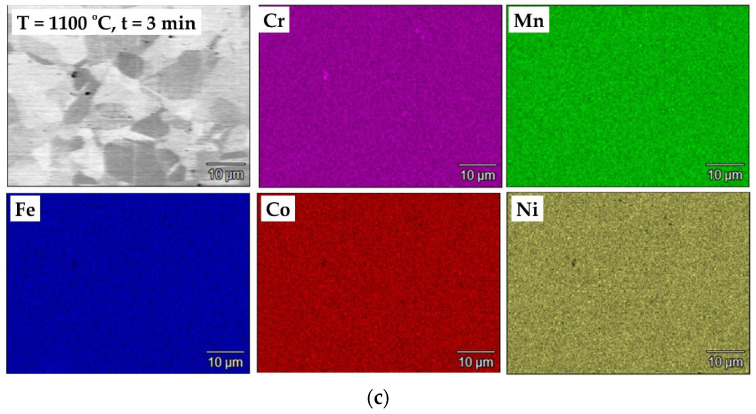
SEM micrographs and corresponding elemental distribution maps (Cr, Mn, Fe, Co, Ni) of the Cantor high-entropy alloy sintered under different conditions: (**a**) T = 1060 °C, t = 5 min; (**b**) T = 1080 °C, t = 5 min; (**c**) T = 1100 °C, t = 3 min.

Elemental distribution maps (Figure 5) obtained using the EDS technique allow for determining the distribution of individual elements present in the chemical composition of the Cantor alloy. The results of the element distribution analysis by the EDS method clearly indicate that all the major alloying elements, such as Cr, Mn, Fe, Co, and Ni, are uniformly distributed within the randomly analyzed areas of the samples. Even at the external surface, no segregation zones, precipitates, non-metallic inclusions, or local deficiencies were detected. The presented analyses confirm the thermal stability of the Cantor alloy. SPS sintering is, however, characterized by difficult control of phenomena at particle boundaries, with electrical pulses potentially causing local temperature spikes beyond those measured by the thermocouple at the die walls. This further substantiates the stability of the alloy’s properties in relation to resistance to high temperatures, although in this case, it is additionally “protected” by the vacuum atmosphere. The heating rate and duration of isothermal holding further benefit microstructural stability, despite intensive phenomena at the boundaries. Notably, manganese, which has high vapor pressure, does not diffuse from the grain interiors to the surface and does not evaporate as can occur in the sintering of manganese steels in gaseous atmospheres [38]. As seen in microstructural images (Figure 4), the only microstructural disruptions here are pores localized at the boundaries of former powder grains used for sintering. The only adverse effect of graphite die use is the need to sandblast the surface after sintering to remove a microns-thick, very brittle layer formed by carbon diffusion, which results in local chromium depletion from grains as it diffuses to the surface and forms chromium carbide.

**Figure 6:**
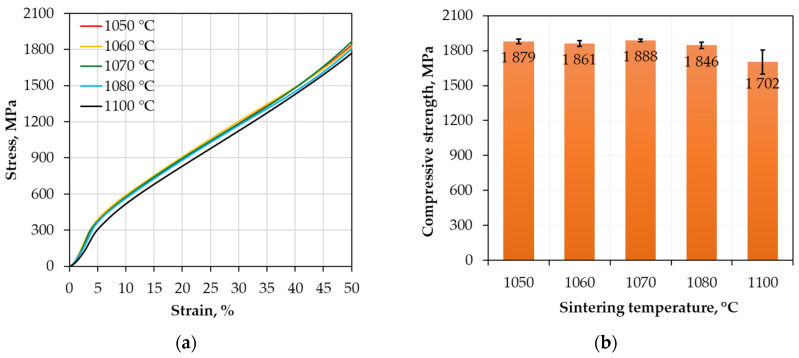
Compression test results of the HEA sintered under different conditions: (**a**) Compression curves; (**b**) Compressive strength.

Analyzing the results obtained from the static compression test, a summary diagram (Figure 6a) was prepared for all tested samples, enabling comparison of the properties of individual sintered compacts. This diagram shows the relationship between true stress (expressed in MPa) and true strain (given in %). A single, representative stress–strain curve for each compact is presented. Based on these results, the compressive strength of the investigated materials was determined, as shown in Figure 6b. The compressive strength was defined as the maximum true stress recorded on the stress–strain curve, with the compression test being stopped when a permanent strain of 50% was reached.

On the graph compression curve progressions for the tested materials (Figure 6a), it is shown that in the initial phase of the deformation process (low strain values), the curves are very similar and mostly overlapping. As the strain increases, the samples sintered at 1050 °C (red line) and 1060 °C (yellow line) stand out, attaining the highest stress values up to approximately 15% strain. At a later stage, from about 40% strain onwards, the sample sintered at 1070 °C (green line) begins to exhibit higher stress values than the others. The sample sintered at 1100 °C (black line) consistently shows the lowest stress values throughout the entire test.

The highest compressive strength was observed for the samples sintered at 1070 °C (1888 MPa), followed closely by those sintered at 1050 °C (1879 MPa) and 1060 °C (1861 MPa). The samples sintered at 1080 °C reached 1846 MPa, while those processed at 1100 °C exhibited the lowest compressive strength of 1702 MPa. These results indicate that moderate sintering temperatures in the range of 1050–1070 °C are most effective in maximizing the compressive strength of the material. This enhanced mechanical per-formance correlates with a refined, fine-grained microstructure and high material density; the average grain size for samples sintered at 1060 and 1070 °C was measured at 8.74 and 9.87 μm, respectively, with densities in the range of 95–96% of theoretical values.

Table 5 presents the average engineering stress values at strain levels of 5% and 25% for samples sintered at different temperatures. The relatively high stress values within this strain range confirm good mechanical strength prior to significant plastic deformation. Except for the sample sintered at 1100 °C, which shows comparatively lower stress likely due to microstructural coarsening or defects induced at this higher temperature, the stresses at 5% and 25% strain remain fairly consistent across other processing temperatures. This indicates mechanical stability of the HEA alloys within the investigated sintering temperature window.

The compressive strength values of CoCrFeMnNi alloy consolidated via Spark Plasma Sintering (SPS) reported in literature typically cluster around 1300 MPa; how-ever, variations arise due to differences in microstructure, including the presence of carbides (such as Cr_7_C_3_) and other secondary phases formed during post-sintering treatments. Our results, obtained on primarily FCC-phase material without significant carbide formation, fall within this expected range and reflect the intrinsic mechanical response of the alloy under the tested conditions. Kumar et al. [31] reported a compressive strength of approximately 1350 MPa for CoCrFeMnNi sintered at 1100 °C under a pressure of 50 MPa. The material exhibited a dominant FCC structure; however, microstructural analysis revealed the presence of Cr_7_C_3_ carbides and trace BCC regions, which may have influenced local mechanical properties. Ruiz-Esparza-Rodriguez et al. [39] compared conventional sintering and SPS for the pure Cantor alloy. The SPS variant achieved compressive strength up to 1280 MPa, with lower porosity and improved grain bonding. Although the FCC structure was dominant, the authors identified oxide inclusions in microregions, particularly in the SPS sample. Peng and Nishimoto [40] reported that CoCrFeMnNi samples processed via SPS reached compressive strength values around 1300 MPa. However, the results were influenced by a nitriding treatment applied to enhance surface hardness.

## 4. Conclusions

This study investigated the influence of spark plasma sintering (SPS) parameters on the densification, microstructural evolution, and mechanical response of the Cantor-type high-entropy alloy. The key findings can be summarized as follows:

- Densification did not follow a linear trend with temperature. The highest density and hardness were achieved at 1050 °C/3 min and 1100 °C/3 min, indicating that local thermo-electrical effects play a decisive role in densification kinetics.
- Pronounced grain growth was observed at 1070–1080 °C/5 min, including abnormal local crystallite growth exceeding 100 µm. Such heterogeneity may deteriorate plasticity and promote stress localization under loading.
- EDS mapping confirmed a uniform distribution of Cr, Mn, Fe, Co, and Ni in all sintered samples, with no detectable segregation or precipitates. Chemical homogeneity is therefore preserved despite variations in sintering parameters.
- The highest compressive strengths (1888 MPa, 1879 MPa, and 1861 MPa) were obtained at 1070 °C/5 min, 1050 °C/3 min, and 1060 °C/5 min, respectively. This demonstrates that sintering at moderate temperatures with either short or slightly longer holding times can enhance strength, provided grain boundary cohesion is maintained.
- Overall, the results show that microstructural homogeneity is more critical than maximizing density alone. Optimizing the balance between densification, grain size control, and elemental uniformity provides a pathway for tailoring HEAs with improved mechanical performance. These insights are directly relevant for the design of HEA components for demanding applications such as aerospace, energy, and tooling industries.

## Figures and Tables

**Table 1:** Chemical composition of the CoCrFeMnNi powder [23].

Fe	Ni	Cr	Mn	Co
19.65	20.67	17.88	19.18	Bal

**Table 2:** Properties of the CoCrFeMnNi powder [23].

D10/μm	D50/μm	D90/μm
21.6	34.4	53.7
Flowability, (s) Bulk density, (g/cm^3^) Microhardness, (HV)	16.2 3.8 155.93 ± 23	

**Table 3:** Sintering parameters of the high-entropy alloy processed by SPS.

Sintering Temperature °C	Time [min]	Heating Rate °/min	Atmosphere
1050	3	300	vacuum
1060	5		
1070	5		
1080	5		
1100	3		

**Table 4:** The effect of sintering temperature on average grain diameter and local large grain size in the investigated samples.

Sintering Temperature °C	Average Grain Diameter [µm]	Local Large Grains (Average Diameter), [µm]
1050	8.78	51.00
1060	8.74	83.94
1070	9.74	68.97
1080	10.59	114.27
1100	9.87	68.05

**Table 5:** The effect of sintering temperature on average stress values for HEA sintered under different condi-tions recorded at 5% and 25% strain.

Sintering Temperature, °C	σ, MPa at ε = 5%	σ, MPa at ε = 25%
1050	390	1050
1060	366	1056
1070	348	1040
1080	370	1038
1100	300	1025

## Data Availability

The original contributions presented in this study are included in the article. Further inquiries can be directed to the corresponding author.
